# Plasma metabonomics study on Chinese medicine syndrome evolution of heart failure rats caused by LAD ligation

**DOI:** 10.1186/1472-6882-14-232

**Published:** 2014-07-09

**Authors:** Qi Qiu, Chun Li, Yong Wang, Cheng Xiao, Yu Li, Yang Lin, Wei Wang

**Affiliations:** 1 Modern Research Center for Traditional Chinese Medicine, Beijing University of Chinese Medicine, Beijing 100029, China; 2 Capital Medical University Beijing An Zhen Hospital, Beijing 100029, China; 3 Basic Medical College, Beijing University of Chinese Medicine, Beijing 100029, China; 4 China-Japan Friendship Hospital, Beijing 100029, China

## Abstract

**Background:**

Chinese medicine syndromes (Zheng) in many disease models are not clearly characterized or validated, and the concepts of Chinese medicine syndromes are confounding and controversial. Metabonomics has been applied to the evaluation and classification of the Chinese medicine syndromes both in clinical and nonclinical studies. In this study, we aim to investigate the evolution of the Chinese medicine syndrome in myocardial infarction induced heart failure and to confirm the feasibility of the Zheng classification by plasma metabonomics in a syndrome and disease combination animal model.

**Methods:**

The heart failure (HF) model was induced by ligation of the left anterior descending coronary artery (LAD) in Sprague–Dawley rats. The rats were divided into the following two groups: the HF model group (LAD ligation) and the sham operated group. GC-MS was used with pattern recognition technology and principal component analysis (PCA) to analyze the plasma samples at 4, 21 and 45 day after operation.

**Results:**

It was determined that the period from 7 to 28 days was the stable time window of ischemic heart failure with qi deficiency and blood stasis syndrome (QDBS), and the qi deficiency syndrome occurred at 1 to 4 days and 45 to 60 days after operation. The results exhibited 5 plasma metabolite changes in the same trend at 4 and 21 day after the LAD operation, 7 at 21 and 45 day, and 2 at 4 and 45 day. No metabolite showed the same change at all of the 3 time points. At day 21 (the QDBS syndrome time point) after operation, 4 plasma metabolites showed the same trends with the results of our previous study on patients with the blood stasis syndrome.

**Conclusions:**

The syndrome diagnosis is reliable in the HF rat model in this study. Plasma metabolites can provide a basis for the evaluation of Chinese medicine syndrome animal models.

## Background

TCM is a complicated system, and the characteristics of the diagnoses and treatments are based on evaluating TCM syndromes, which are becoming more common in clinical practice and have a history of more than one thousand years. With the development of science, TCM faces severe challenges and suffers from insufficient modern research because the lack of scientific and technological approaches restricts the development of TCM [1,2]. Animal models play an important role in basic research and in the evaluation of drug effects. To reveal the biological basis of TCM syndromes and perform an objective evaluation of Chinese herbs, animal models that have the features of the disease and syndromes should be established.

Zheng classification (Bian Zheng) is a traditional diagnostic method to categorize patients’ syndromes based on their presenting conditions [3]. A combination of Zheng classification and biomedical diagnoses has become a common model in TCM diagnostics in clinical practice [3]. A novel analytical technique, metabonomics (or metabolomics), adopts a ‘top-down’ strategy to reflect the function of organisms from the terminal symptoms of the metabolic network and to understand the metabolic changes of a complete system caused by interventions in the holistic context [1]. Metabonomics realistically reflects the overall environment of various characteristic changes in humoral metabolites and reveals the overall human metabolic network and the laws governing metabolic changes under the influence of medicine [4]. This property is consistent with the holistic philosophy of TCM and might provide an opportunity to express scientifically the meaning of evidence-based Chinese medicine, such as Chinese medicine syndromes (CMS), preventive treatment, the action of Chinese medicine, Chinese medical formulas (CMF) and acupuncture efficacy [1,5]. In a clinical study, the metabonomics method was applied to study clinical urine samples in patients with suboptimal health with different syndromes [6]. In nonclinical studies, the metabonomics method has been applied in discussing the syndrome of the stagnation of liver Qi and spleen deficiency and has assisted in revealing the biological changes in the rats with chronic restraint stress-induced syndrome of stagnation of the liver Qi and spleen deficiency [5,6]. The analysis of plasma metabonomics was applied in mini-swines with qi deficiency and blood stasis syndrome from chronic myocardial ischemia [7] and in research on the evaluation of the fever syndrome model in rats [8]. In a combined clinical and nonclinical study, the metabonomics method was used to analyze clinical and animal plasma samples in coronary heart disease with the blood stasis syndrome [9].

Gas chromatography (GC)- Mass Spectrometry (MS) with electron ionization remains the technique of choice for the metabolic profiling of the polar intermediates of primary metabolism because of its high sensitivity, its ability to better discriminate the compounds in the gas phase than the liquid phase and the ability to identify unknown compounds based on fragmentation patterns and the existence of well-established analysis databases for metabolite identification [10,11]. The high sensitivity of GC-MS could allow for the quantification of metabolites present in small amounts and might indicate subtle physiological differences between samples that might be undetectable in an LC-MS profile [12,13]. For these advantages to occur, the GC-MS metabolic profile must be appropriately corrected for experimental biases.

This study used time course plasma metabonomics analyses based on GC-MS with pattern recognition technology, principal component analysis (PCA) and the combined disease/syndrome animal model of myocardial infarction (MI) induced heart failure (qi deficiency/qi deficiency and blood stasis) in rats to screen for possible differences in the characteristics of metabolites and characteristic pattern combinations and to investigate the material basis of the evolution of syndromes in HF rats.

## Methods

### Animals and groups

All the animal experiments were performed in accordance with the China Physiological Society’s “Guiding Principles in the Care and Use of Animals” with the approval of the Animal Care Committee of Beijing Medical Center. A total of 60 male SD rats (weighing 240 ± 10 g) of SPF grade were selected (purchased from Beijing Vital River Laboratory Animal Technology Co. Ltd. Beijing, China) for this study. The rats were housed in a standard animal room with food and water provided ad libitum under controlled conditions of humidity and temperature (25 ± 1.2°C) with a 12 h light: 12 h dark schedule.

### HF model preparation

The HF model was established as described previously [14]. Pentobarbital-anesthetized rats were restrained on the operating table. The thoracic cavity was opened to expose the heart, and the left coronary artery (LAD) was ligated. In the sham operated group, sutures were passed under the LAD without ligation. The animals were housed routinely following surgery. At 4, 21 and 45 d after the operation, the animals were anesthetized using isoflurane (Abraxis BioScience, Richmond Hill, Ontario, Canada) following an overnight fast, with eight rats examined at each time point. The blood samples were obtained via tail puncture, and the plasma was isolated after centrifugation of the blood samples at 1700 × g at 4°C within the first hour after blood collection. The plasma samples were stored at −80°C until the plasma metabonomics analyses of heart failure with qi deficiency and blood stasis (QDBS) were performed.

### Evaluation of the disease and TCM syndrome

The ECG, physical signs, echocardiography, blood studies and colorimetric analysis of images including auricle, plantar and tongue were used to evaluate qi deficiency and blood stasis syndrome in model animals.

#### ECG

The surface electrocardiograms (ECG) were obtained by FX7200 ECG (Fukuda, Japan) at day 3 after operation, in order to determine the myocardial infarction size in MI rats.

#### Physical signs

The rats’ body weight, respiratory rate in the resting state, and other physical signs were obtained at 4, 7, 14, 21, 28, 45, 60 day after surgery.

#### Echocardiography

Transthoracic echocardiography was performed using ACUSON Sequoia 512 with 15L8W-S transducer (Siemens, Germany). The rats were anesthetized with pentobarbital sodium (1%, 20 mg/kg IP) at 4, 7, 14, 21, 28, 45 and 60 day after surgery. The rats were kept on a heated platform for the acquisition of 2-dimensionally guided M-mode images at the tip of the papillary muscles.

#### Blood studies

Blood (sodium citrate 1:9) was collected from the rats at 4, 7, 14, 21, 28, 45 and 60 days after surgery. The blood was divided into 4 groups: plasma, whole blood, and platelet-rich plasma, and the detected indexes including hematocrit, platelet aggregation rate (Platelet Aggregometer DIC PA-3210, JAPAN), plasma viscosity, blood viscosity (Rotary Viscometer LBY-W6, Percil, CHINA) and fibrinogen. The methods followed the reference [4].

#### Colorimetric analysis of images of the auricle, plantar and tongue

After taking pictures of the auricle, foot plant and tongue surface in 20 rats (10 in each group) at every time point after the operation, the color indexes were analyzed using Photoshop software. All the pictures were acquired with the correction of a Casmatch color and size-matching sticker (BEAR medic corporation, Japan) following the instructions.

### GC–MS analysis

#### Chemicals

The following chemicals were used in the GC–MS analysis: chlorotrimethylsilane, TMCS from Fluka Inc. (CA, USA), methyl cyanides from Fisher Co., Ltd. (CA, USA), N-Methyl-N- (trimethylsilyl)-trifluoroacetamide (MSTFA) from Fluka Inc. (CA, USA) and methoxylamine hydrochloride from Fisher Co., Ltd. (CA, USA).

#### Sample preparation

At 4, 21 and 45 days after operation, the animals were anesthetized using isoflurane (Abraxis BioScience, Richmond Hill, Ontario, Canada) following an overnight fast, with eight rats examined at each time point. The blood samples were obtained via tail puncture, and the plasma was isolated after centrifugation of the blood samples at 1700 × g at 4°C within the first hour after the blood collection. The plasma samples were stored at −80°C until the plasma metabonomics analyses of heart failure with qi deficiency and blood stasis (QDBS) were performed.

Plasma preparation was performed as described [7]. Methyl cyanide (250 μL) was mixed into 100-μL aliquots of the plasma samples at each time point. The samples were centrifuged at 12100 × g for 10 min after being placed in an ice bath for 10 min. A 250-μL aliquot of the supernatant was transferred to an Eppendorf tube with a pierced cap. The samples were dried in a vacuum centrifuge dryer and in a freeze dryer. For derivatization, 50 μL of methoxylamine hydrochloride in pyridine (15 mg/mL; the first derivatizing agent) were added to the samples. The mixture was incubated at 70°C for 60 min, after which the second derivatizing agent, consisting of 50 μL of a mixed solvent (MSTFA: MCS = 100: 1, v/v), was added. The mixture was incubated at room temperature for 60 min. One hundred microliters of n-heptane containing docosanoic acid (0.10 mg/mL), was added as an internal standard, and the solution was mixed and centrifuged (1812 × g, 10 min). The supernatant was transferred to a micro-injector tube, and a 1-μL sample was injected in split mode (25:1, v/v).

#### Sample analysis

Chromatography was performed using the TRACE GC Ultra-DSQ II GC/MS system (Thermo Fisher Scientific, Massachusetts, USA) using an RTx-5 capillary column (30 m × 0.250 mm × 0.25 μm), the Xcalibur workstation and the NIST spectral library [7]. The injection temperature was 270°C, and the helium gas flow rate through the column was 1 ml/min. The column temperature was isothermally maintained at 80°C for 5 min and raised to 300°C at a rate of 10°C/min. The temperature was then isothermally maintained at 300°C for 5 min. The temperatures of the transfer line and the ion source were 280°C and 230°C, respectively. The ions were generated at an electron impact (EI) energy of 70 kV, and 20 scans/s were recorded over the mass range of 45–550 m/z. The column temperature program was in Table 1.

**Table 1:** The column temperature program

**Time (min)**	**Heating rate (°C /min)**	**Temperature (°C)**
0-2	0	80
3-12	10	180
13-15	5	230
26-27	25	290
27-35	0	300

#### GC–MS data acquisition and alignments

The primitive GC/MS data mapping files were imported directly into MATLAB (MathWorks, Inc., USA) script and preprocessed. This process can be divided into steps including denoising smoothing, baseline correction, peak calibration, split window, overlapping peak identification and peak area integration. A three-dimensional matrix was obtained, and the three-dimensional coordinates were as follows: the characteristics of the compound index (expressed as the mass-to-charge ratio), the sample name, and the normalized peak area. According to the GC-MS total ion chromatogram, the peak retention time of each selected peak, all of the test compound spectra and retention indices were compared with the spectra and retention indices of the U.S. National Institute of Standards and Technology (NIST) Library (2008) for peak identification; the results were considered reliable because more than 80% of the results were identified based on these comparisons. The peak area data were obtained and expressed as a percentage based on the peak area metabolite levels.

#### Multivariate analysis of the samples

The intensities of each peak were normalized based on the creatinine concentrations determined for each animal. The generated peak lists were imported into SIMCA-P (Ver.11.5) (Umetrics, Sweden) for a multivariate statistical analysis. A principal component analysis (PCA) was performed to identify the relationships between the various groups of multivariate data in terms of the similarities or differences. In the t test mining conducted to make comparisons between the groups, P <0.05 indicates a significant difference. The principal component scores were analyzed using analysis of variance (ANOVA) and multiple comparison tests; P <0.05 was considered statistically significant.

## Results

### The syndrome diagnosis

The results of the body weight, respiratory rate, blood studies, echocardiography and colorimetric analysis of rats are given in Additional file 1: Table S1.

#### ECG

Pathological Q waves were observed in 6–8 leads on a surface electrocardiogram in the model animals. No significant arrhythmias were found in the experimental period.

#### Physical signs

There were no significant differences on the body weight between model and sham rats at the same time point. The respiratory rates of model rats were slower at each time point in the resting state when comparing to the sham operation rats. During the period 1 to 3 days after operation, the model rats and the sham operated rats acted slowly in activity and response to external stimuli; 4 to 6 days after the operation, the model rats were sensitive to external stimuli, mental stress, irritation, fear, showing hair erection and lack of luster, and the sham rats’ performances gradually returned to normal; 7 to 60 days after surgery, the model animals appeared to show hair erection and lack of luster, significantly reduced activity, gradually slower responses to external stimuli, and weakness, whereas the sham rats’ performances were normal.

#### Echocardiography

The left ventricular ejection fraction (LVEF) and left ventricular fractional shortening (LVFS) were significantly decreased at different time points after LAD (P < 0.05, P < 0.01) in the model rats compared with the sham rats. The results showed a segmental dysfunction of the left ventricular.

#### Blood studies

Compared with the sham operated group, the PA%, AV (50S-1, 200S-1), RV (10S-1, 50S-1) at 7 days; the PCV%, PA%, AV (10S-1, 50S-1) and RV 10S-1 at 14 days; the PCV%, AV (10S-1, 50S-1, 200S-1) and RV 10S-1; and PCV%, AV 10S-1 were increased significantly (P < 0.05 or P < 0.01) in the model group.

#### Colorimetric analysis of images of the auricle, plantar and tongue

Compared with the sham operated group, the plantar R-value was decreased (P < 0.05) at 7 days after operation, and the three color indexes (including the R, G and B values) were decreased at 14, 21 and 28 days (P < 0.05, P < 0.01); the tongue R-value (p < 0.01) and G-value (p < 0.05) were decreased at 14 days, and the tongue B-value (p < 0.01) was increased significantly in the model rats. During the period 7 to 28 days after surgery, the auricle in the model rats had visible varicose veins and purple color, and the ear skin became pale. The auricle G-value was decreased significantly (p < 0.05) compared with the sham rats.

According to the blood stasis diagnostic criteria published by the China Association of Integrative Medicine, Professional Committee on Blood Circulation in 1986 and the TCM deficiency syndrome reference standard published by the National Integrative Deficiency & Geriatrics Research Committee in May 1986, combined with the five aspects above, the syndromes of the myocardial infarction caused heart failure rats were diagnosed. The period from 7 to 28 days is the stable heart failure with qi deficiency and blood stasis (QDBS) time window, and the two periods, 1–4 days and 45–60 days after the operation were diagnosed as the qi deficiency syndrome. The blood samples from both groups were analyzed by GC-MS at 4, 21, and 45 day after operation in 3 syndrome time windows respectively.

### The results of GC/MS analysis

#### Typical GC-MS total ion chromatograms for plasma samples at each time point following the operation

**Figure 1:**
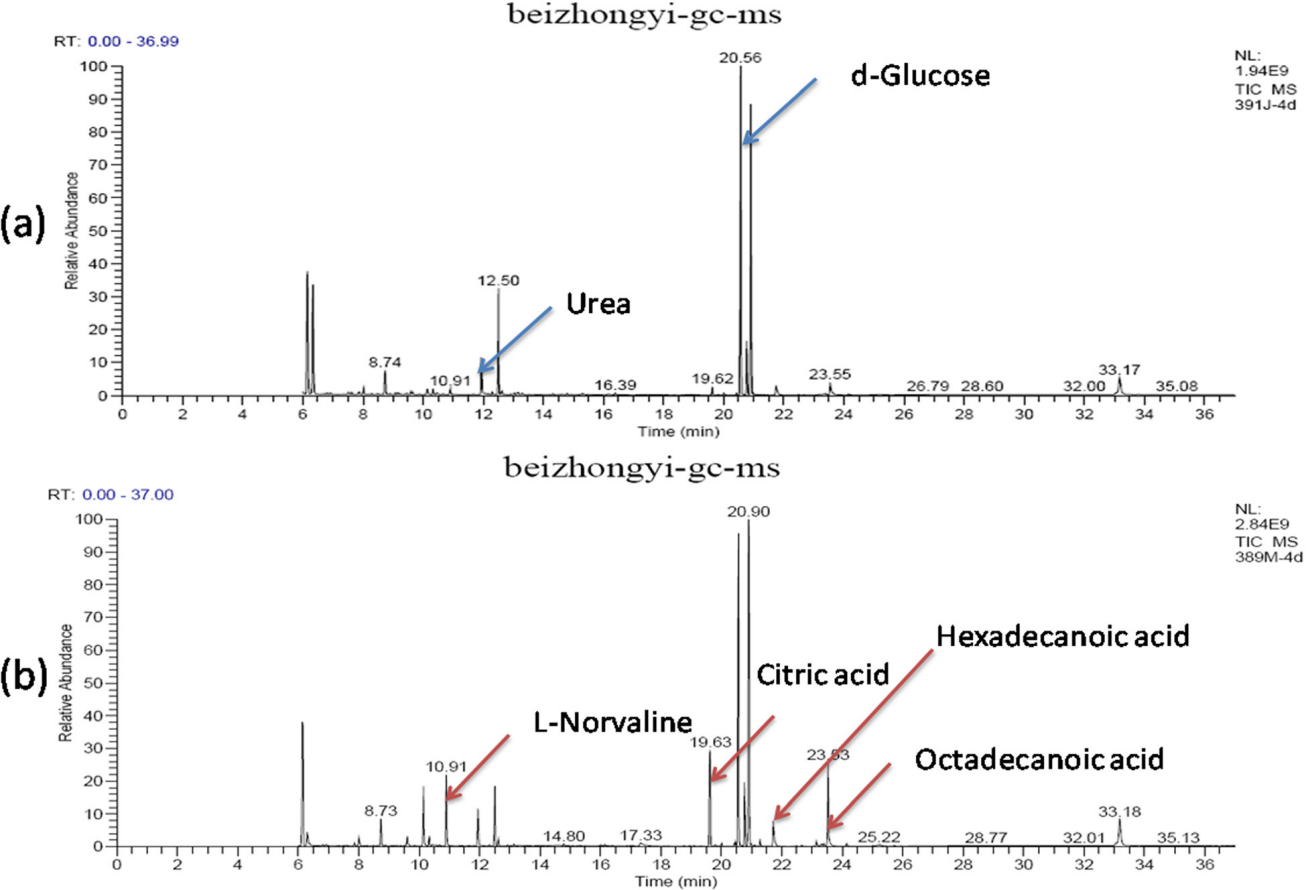
**GC spectra obtained in the sham operated and model groups 4 days after the operation. (a)**: sham group, **(b)**: model group.

**Figure 2:**
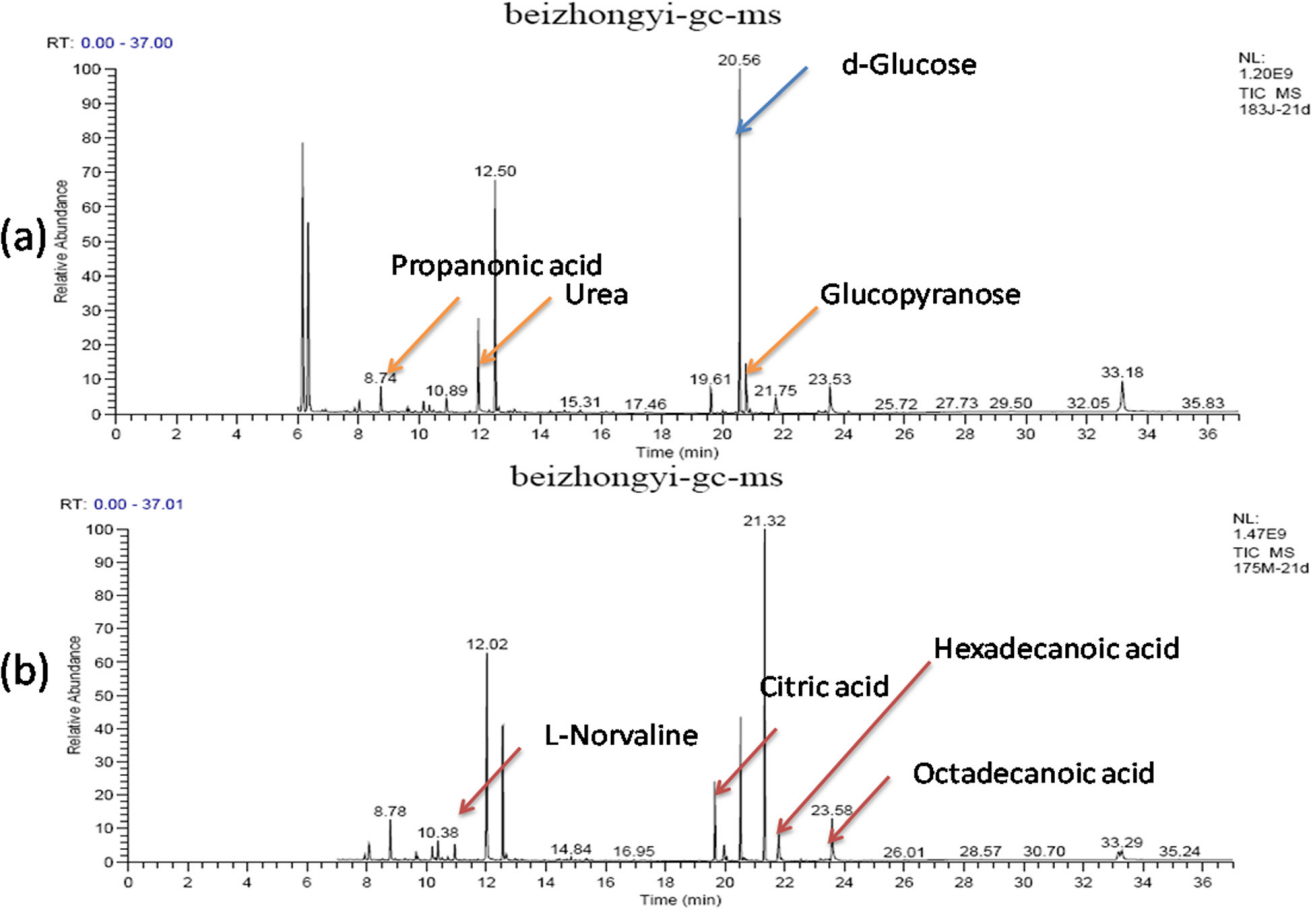
**GC spectra obtained in the sham operated and model groups 21 days after the operation. (a)**: sham group, **(b)**: model group.

**Figure 3:**
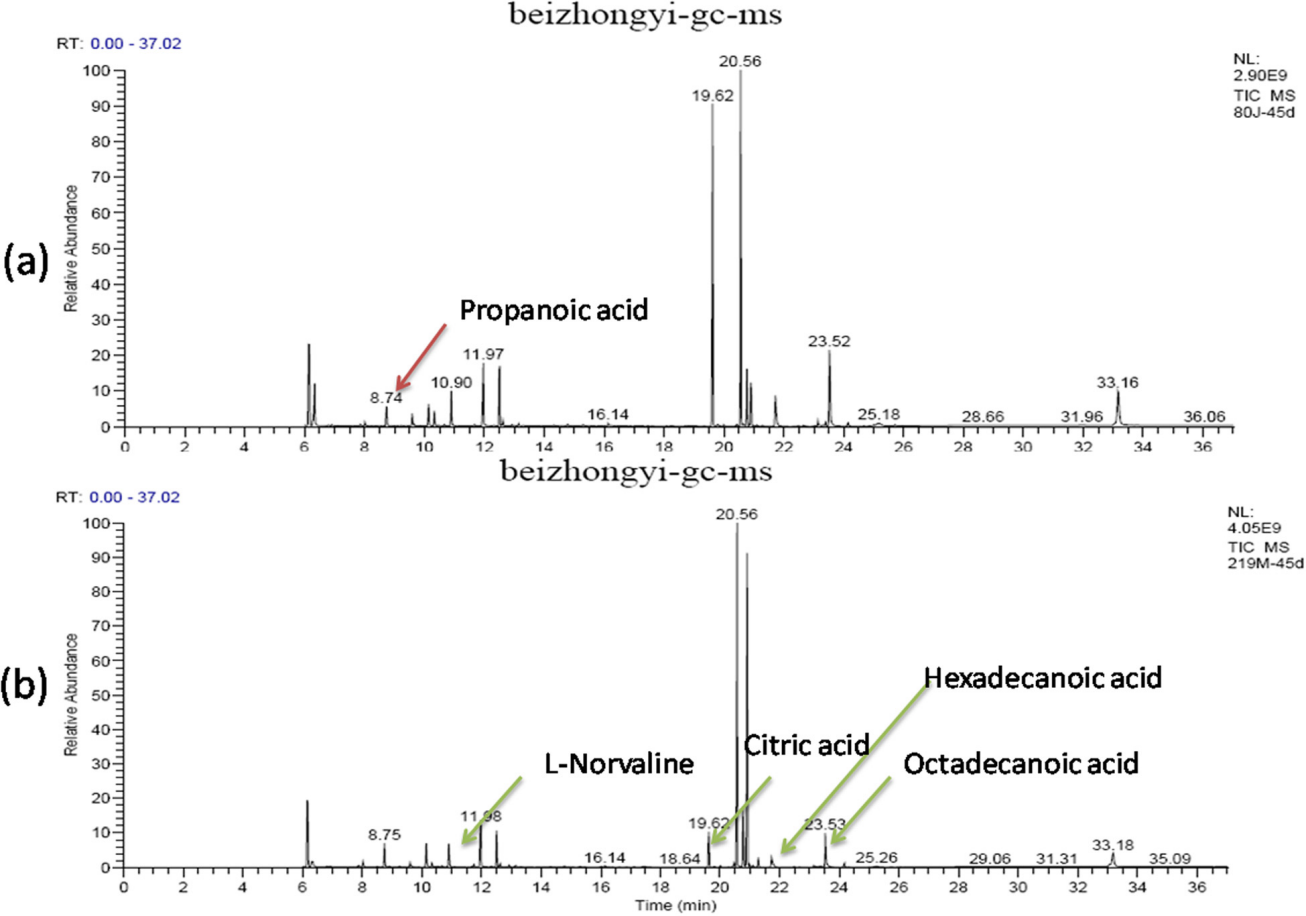
**GC spectra obtained in the sham operated and model groups 45 days after the operation. (a)**: sham group, **(b)**: model group.

The results of plasma samples analysis from the model and sham rats are shown in Figures 1, 2 and 3. Based on the automatic peak identification procedures and standard database, this study identified a variety of metabolites, including small organic molecules such as amino acids, and other compounds such as liposome components. In the total ion chromatogram, the peaks showed a clear difference between the model and sham groups.

At day 4 after operation, metabolites changes were observed in model rats after compared to sham ones. The increases were observed in the levels of energy-related metabolites such as citric acid and cAMP; amino acid metabolites such as L-norvaline and glycine; sugar metabolites such as à-D-glucopyranoside; and lipid metabolites such as dodecanoic acid, hexadecanoic acid and octadecanoic acid. Decreases were observed in the levels of amino acid metabolites such as acetamide, trimethylsilyl amine, alanine, L-valine, urea, L-serine; the glucose metabolites such as d-glucose, d-galactose and glucitol; and the fatty acid metabolites such as acetic acid, propanoic acid and butanoic acid. These results are shown in Figure 1.At day 21 after operation, the metabolites changes in model group including the increases in energy-related metabolites such as citric acid; amino acid metabolites such as acetamide, ethylbis (trimethylsilyl) amine, alanine, L-norvaline, L-valine, urea and L-serine; the glucose metabolite à-D-glucopyranoside; and lipid metabolites such as acetic acid, propanoic acid, butanoic acid, dodecanoic acid, hexadecanoic acid and octadecanoic acid; and decreases in energy-related metabolites such as cAMP, amino acid metabolites such as glycine and L-proline. These results are shown in Figure 2.At day 45 after operation, the model group showed metabolic changes including increased levels of amino acid metabolites such as ethylbis (trimethylsilyl) amine and L-serine and lipid metabolites such as propanoic acid and butanoic acid; and decreases in the levels of energy-related metabolites such as citric acid and cAMP; amino acid metabolites such as acetamide, alanine, L-norvaline, L-valine, urea and L-serine; the glucose metabolite à-D-glucopyranoside; and fatty acid metabolites such as hexadecanoic acid and octadecanoic acid. These results are shown in Figure 3.

#### Pattern recognition and multi-dimensional statistical analysis

Principal components analysis (PCA) was conducted to achieve a reduction of data dimensionality, exclude overlapping information and convert the original variables into a small number of new variables that were linear combinations of the original variables. These new variables were employed to characterize the structure of the original data without a loss of information [15].

**Figure 4:**
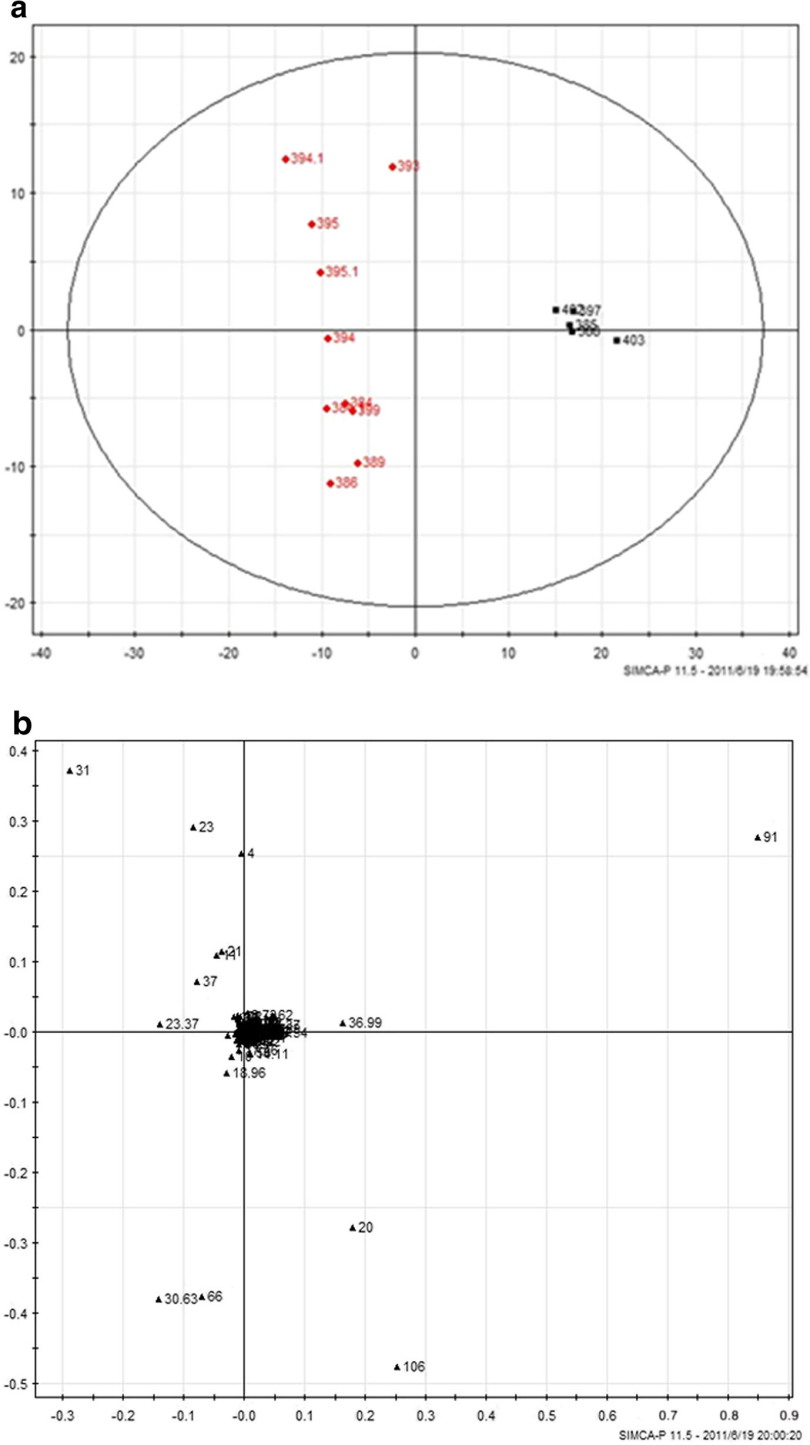
**The PCA score plot and load chart for the GC-MS spectra obtained for the two groups 4 days after the operation.** The clusters of the sham and model group metabolites are separated by the t1 axis (minor axis), and the PCA score covered 90% of the total information. **(a)**: Principal component score plots; **(b)** loading plots. (red square symbol): Sham operated group; (black square symbol): Model group.

**Figure 5:**
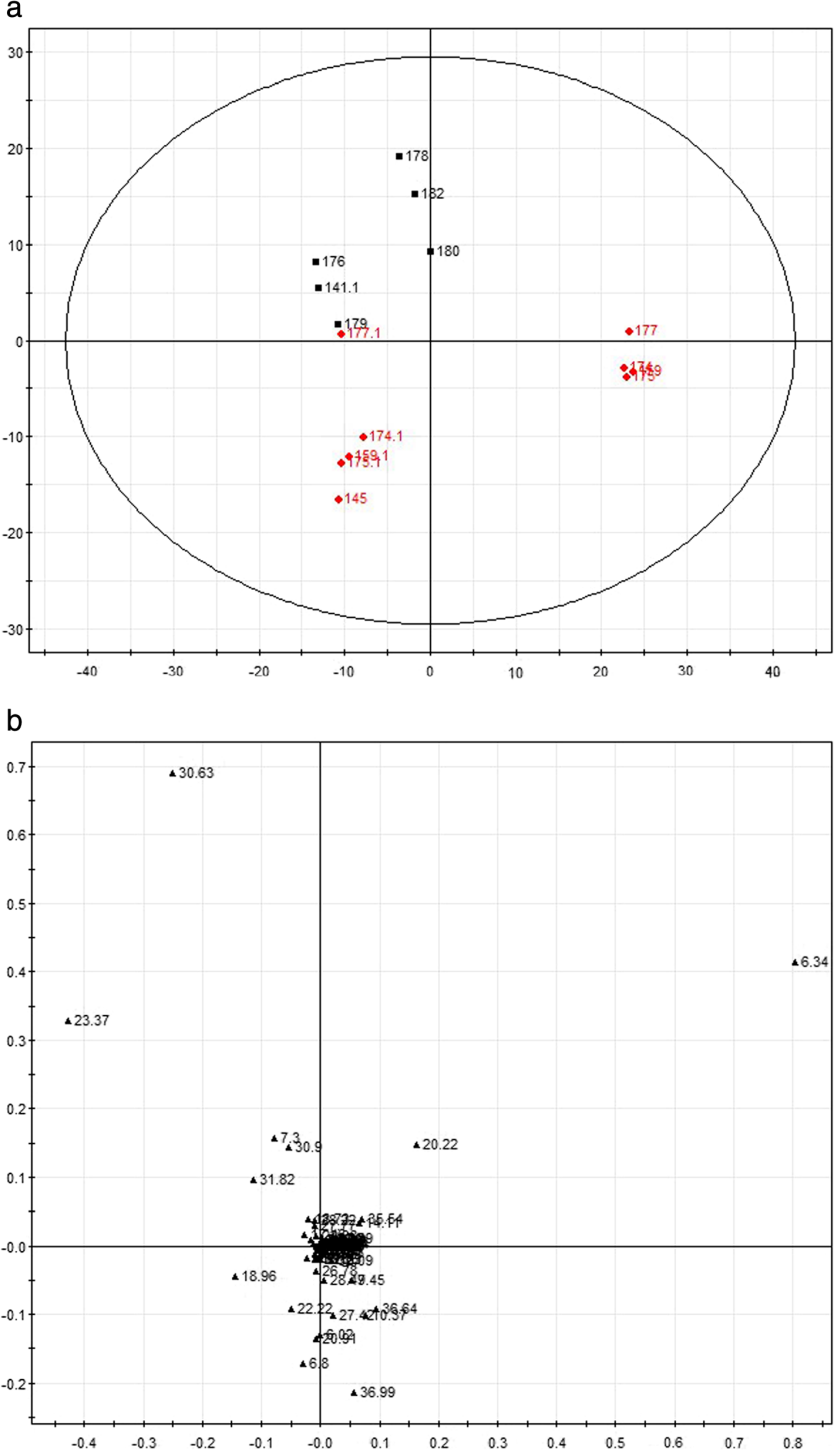
**The PCA score plot and load chart for the GC-MS spectra obtained for the two groups 21 days after the operation.** The clusters of the sham and model group metabolites are separated by the t2 axis (minor axis), and the PCA score covered 90% of the total information. **(a)**: Principal component score plots; **(b)** loading plots. (red square symbol): Sham operated group; (black square symbol): Model group.

**Figure 6:**
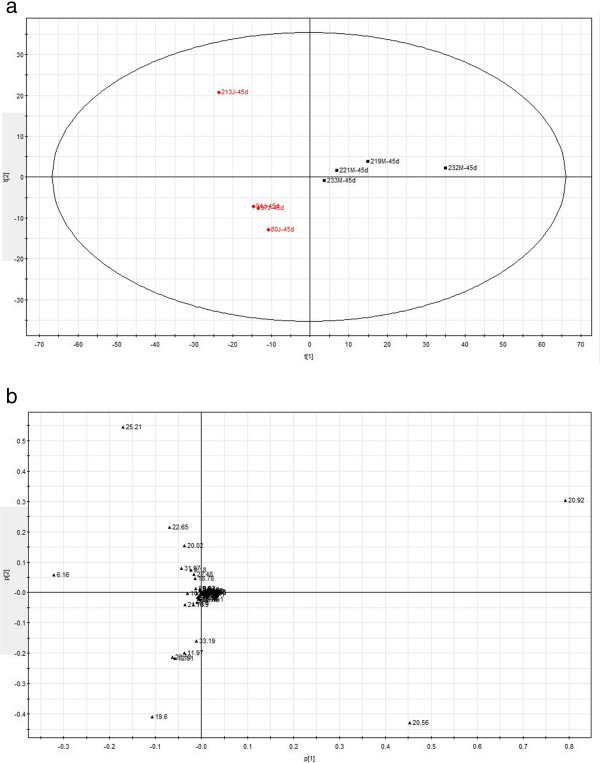
**The PCA score plot and load chart for the GC-MS spectra obtained for the two groups 45 days after the operation.** The clusters of the sham and model group metabolites are separated by the t1 axis (minor axis), and the PCA score covered 90% of the total information. **(a)**: Principal component score plots; **(b)** loading plots. (red square symbol): Sham operated group; (black square symbol): Model group.

In this study, the PCA method was applied to compare the GC spectra obtained in model and sham rats, and the results are shown in Figures 4, 5 and 6. The composition of the plasma displayed stability in all of the samples, and most of the identified compounds were clustered in a specific area. The metabolites that showed clear differences and contributed markedly to the PCA classification included citric acid, cAMP, stearic acid, palmitic acid, lauric acid, urea, norvaline and α-D-glucose glycoside compounds. The representative metabolites in the plasma and the changes in the GC peak signal intensity for the various metabolites are listed in Tables 2, 3 and 4.

**Table 2:** Plasma metabolites detected in the sham-operated and model animals 4 days after the operation

**Metabolic pathway**	**RT (min)**	**Metabolites**	**Sham-operated (×10**^**9**^**)**	**Model (×10**^**9**^**)**	***P *** **value**
TAC	19.60	Citric acid	1.06 ± 0.721	4.54 ± 1.282^▲▲^	0.000
	24.18	cAMP	0.20 ± 0.035	0.26 ± 0.036^▲^	0.033
Amino acid metabolism	6.90	Acetamide	0.43 ± 0.084	0.027 ± 0.066^▽▽^	0.001
	8.01	Ethylbis(trimethylsilyl)amine	0.85 ± 0.025	0.52 ± 0.130^▽▽^	0.006
	9.45	Alanine	0.90 ± 0.230	0.52 ± 0.122^▽▽^	0.000
	10.91	L-Norvaline	0.83 ± 0.428	2.38 ± 0.0932^▲▲^	0.000
	11.67	L-Valine	0.18 ± 0.056	0.08 ± 0.046^▽▽^	0.002
	11.97	Urea	4.75 ± 1.096	3.55 ± 0.829^▽^	0.022
	13.14	Glycine	0.324 ± 0.068	0.69 ± 0.174^▲▲^	0.000
	14.11	L-Serine	1.95 ± 0.234	1.36 ± 0.301^▽▽^	0.003
	16.15	L-Proline	0.42 ± 0.038	0.42 ± 0.045	0.941
Glucose metabolism	20.57	d-Glucose	22.78 ± 6.987	15.97 ± 3.549^▽^	0.013
	20.91	d-Galactose	27.47 ± 7.200	14.28 ± 3.173^▽▽^	0.002
	15.30	à-D-Glucopyranoside	0.27 ± 0.117	0.42 ± 0.137^▲^	0.026
	20.95	glucitol	22.79 ± 0.683	0.00 ± 0.000^▽^	0.026
Fatty acid metabolism	8.77	Lactic acid	1.93 ± 0.319	1.36 ± 0.325^▽▽^	0.005
	9.22	Acetic acid	0.24 ± 0.017	0.18 ± 0.139^▽▽^	0.009
	14.80	Butanoic acid	0.47 ± 0.166	0.73 ± 0.192^▲▲^	0.009
	17.48	Dodecanoic acid	0.00 ± 0.000	0.70 ± 0.562^▲▲^	0.001
	21.73	Hexadecanoic acid	1.68 ± 0.640	2.86 ± 1.387^▲^	0.026
	23.54	Octadecanoic acid	4.03 ± 1.332	6.36 ± 1.847^▲^	0.017

Note: ^▲^increased significantly in model group, P < 0.05 compare with the Sham Group, ^▲▲^P < 0.01 compare with the Sham Group; ^▽^decreased significantly in model group, P < 0.05 compare with the Sham Group, ^▽▽^P < 0.01 compare with the Sham Group.

**Table 3:** Plasma metabolites detected in the sham-operated and model animals 21 days after the operation

**Metabolic pathway**	**RT (min)**	**Metabolites**	**Sham-operated (×10**^**9**^**)**	**Model (×10**^**9**^**)**	***P *** **value**
TAC	19.60	Citric acid	1.92 ± 1.909	4.33 ± 1.240^▲^	0.026
	24.18	cAMP	0.27 ± 0.092	0.10 ± 0.052^▽^	0.006
Amino acid metabolism	8.01	Ethylbis(trimethylsilyl)amine	0.79 ± 0.143	1.29 ± 0.437^▲^	0.048
	9.60	Alanine	0.64 ± 0.037	1.08 ± 0.311^▲^	0.036
	10.94	L-Norvaline	1.26 ± 0.616	2.28 ± 0.687^▲^	0.029
	11.67	L-Valine	0.12 ± 0.032	0.29 ± 0.117^▲^	0.014
	12.00	Urea	8.05 ± 1.569	13.41 ± 2.833^▲▲^	0.008
	13.17	Glycine	0.57 ± 0.247	0.27 ± 0.057^▽^	0.028
	14.11	L-Serine	1.36 ± 0.301	1.76 ± 0.476^▲▲^	0.001
	16.94	L-Proline	0.57 ± 0.232	0.11 ± 0.027^▽▽^	0.005
Glucose metabolism	21.31	Glucopyranose	0.22 ± 0.050	20.64 ± 1.144^▲▲^	0.000
	22.52	Glucopyranoside	0.07 ± 0.017	0.16 ± 0.046^▲^	0.023
Fatty acid metabolism	8.77	Lactic acid	1.48 ± 0.036	2.62 ± 0.345^▲▲^	0.000
	9.25	Acetic acid	0.29 ± 0.073	0.47 ± 0.075^▲▲^	0.004
	14.80	Butanoic acid	0.39 ± 0.065	0.80 ± 0.212^▲▲^	0.009
	17.48	Dodecanoic acid	0.40 ± 0.050	1.02 ± 0.465^▲^	0.021
	21.73	Hexadecanoic acid	1.87 ± 0.320	2.67 ± 0.398^▲▲^	0.006
	23.54	Octadecanoic acid	3.58 ± 0.965	5.49 ± 0.670^▲▲^	0.004
	25.99	Octadecatrienoic acid	0.07 ± 0.036	0.02 ± 0.117^▽^	0.022
	29.84	Monolinoleoylglycerol	1.13 ± 0.562	0.38 ± 0.201^▽^	0.040

Note: ^▲^increased significantly in model group, P < 0.05 compare with the Sham Group, ^▲▲^P < 0.01 compare with the Sham Group; ^▽^decreased significantly in model group, P < 0.05 compare with the Sham Group, ^▽▽^P < 0.01 compare with the Sham Group.

**Table 4:** Plasma metabolites detected in the sham-operated and model animals 45 days after the operation

**Metabolic pathway**	**RT (min)**	**Metabolites**	**Sham-operated (×10**^**9**^**)**	**Model (×10**^**9**^**)**	**P value**
TAC	19.6	Citric acid	9.14 ± 7.010	1.91 ± 1.550^▽^	0.020
	24.16	cAMP	0.44 ± 0.200	0.13 ± 0.236^▽^	0.040
Amino acid metabolism	8.01	Ethylbis(trimethylsilyl)amine	0.37 ± 0.124	0.69 ± 0.134^▲^	0.016
	9.45	Alanine	0.67 ± 0.250	0.41 ± 0.148^▽^	0.015
	10.15	Silanamine	2.34 ± 0.423	1.45 ± 0.537^▽^	0.041
	10.91	L-Norvaline	2.14 ± 0.473	1.02 ± 0.909^▽^	0.047
	11.67	L-Valine	0.29 ± 0.117	0.12 ± 0.032^▽^	0.014
	12.00	Urea	4.775 ± 0.851	6.96 ± 1.963^▲^	0.086
	13.17	Glycine	0.52 ± 0.154	0.24 ± 0.147^▽^	0.024
	14.11	L-Serine	1.17 ± 0.511	1.67 ± 0.266^▲▲^	0.001
	16.15	L-Proline	0.51 ± 0.104	0.36 ± 0.047^▽^	0.036
Glucose metabolism	20.02	Ribitol	0.02 ± 0.053	0.35 ± 0.192^▲▲^	0.002
	22.52	Glucopyranoside	0.16 ± 0.046	0.07 ± 0.017^▽^	0.023
Fatty acid metabolism	8.73	Lactic acid	1.27 ± 0.102	1.69 ± 0.319^▲^	0.039
	14.80	Butanoic acid	0.38 ± 0.080	0.52 ± 0.076^▲^	0.028
	17.48	Dodecanoic acid	0.56 ± 0.811	0.37 ± 0.209	0.550
	21.78	Hexadecanoic acid	2.45 ± 0.483	1.30 ± 0.707^▽^	0.023
	23.19	Heneicosane	0.74 ± 0.256	0.06 ± 0.181^▽▽^	0.000
	23.53	Octadecanoic acid	6.07 ± 0.927	4.28 ± 1.103^▽^	0.047
	28.32	Octadecatrienoic acid	1.25 ± 0.149	0.48 ± 0.309^▽▽^	0.002

Note: ^▲^increased significantly in model group, P < 0.05 compare with the Sham Group, ^▲▲^P < 0.01 compare with the Sham Group; ^▽^decreased significantly in model group, P < 0.05 compare with the Sham Group, ^▽▽^P < 0.01 compare with the Sham Group.

**Figure 7:**
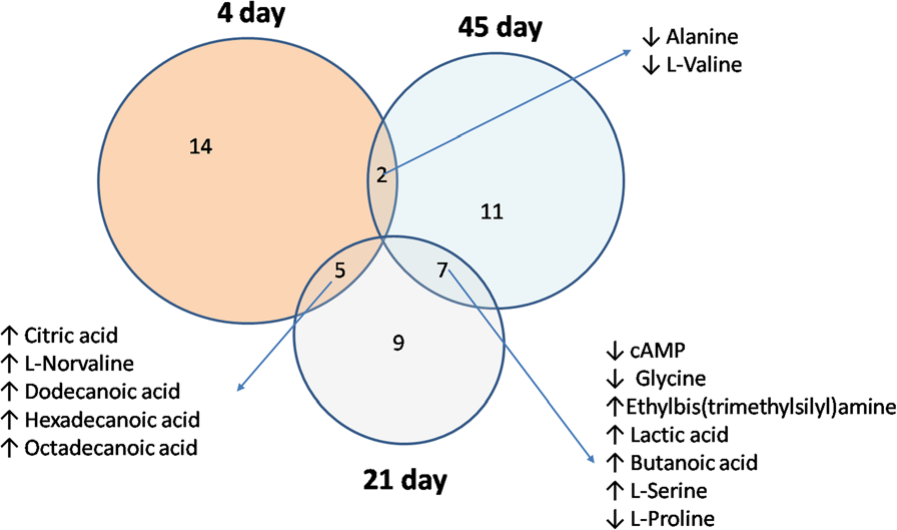
**The conclusion of plasma metabolite changes at each time point after operation.** Venn diagram showing the distribution of metabolites altered in different time point.

To better conclude the results of GC-MS analysis, we compared metabolic alterations in different time point (Figure 7). In addition to the 14, 9, and 11 metabolites signature that distinguished qi-deficiency and QDBS syndrome from non-qi deficiency and non-QDBS respectively, 14 additional metabolites were detected in all the 3 time point with different change trend. There were 5 metabolites showed the same change trend between 4 and 21 day, 7 between 21 and 45 day, and 2 between 45 and 4 day, which reflected the evolution of syndromes.

## Discussion

Currently, combination of Zheng classification and biomedical diagnosis becomes a common model in TCM diagnostics in both clinical and nonclinical practice. In our previous study, the Data mining technology based on the Shannon entropy mutual information was used to analysis the complicated correlations of the statistical distribution of coronary heart disease syndromes associated physical and chemical indexes. We founded that echocardiography and hemorheology was the most closely correlated index with qi-deficiency and blood-stasis syndrome respectively [16]. In clinical research, Li H holds that LVEF can reflect the severity of heart failure, and could be taken as the beneficial objective and quantitative indices for syndrome pattern differentiation [17]. In nonclinical research, echocardiography and respiratory rate was used to evaluate the effect of Qiliqiangxin capsule on cardiac function in rats with chronic heart failure of heart qi deficiency [18]. The indexes such as tongue appearances, blood hyperviscosity platelet aggregation rate and fibrinogen were widely use in the evaluation of blood stasis in both clinical and nonclinical research [19-21]. Moreover, LARS showed that there was a significant correlation between color indexes and the results of hematocrit and platelet aggregation rate in our previous study [22].

Recently, many disease models are not clearly characterized or validated in the Chinese medicine syndrome and metabonomics are confounding and controversial concepts. Wang X believes that trying to connect western medicine and traditional oriental medicine using the metabonomics approach is probably premature at this stage because there is no definitive clinical characterization of traditional oriental medicine [1]. Blood is the most important substance that comes in contact with tissues [23], which contains many types of nutrients such as inorganic salts, sugars, amino acids, lipids and hormones that directly reflect the changes occurring in the body from biological activities [24].

In the present study, the characteristics of qi deficiency were shown in the entire time window from 4 to 60 days after the operation, with a significantly slowed breathing rate and decreased heart function, which are closely related to energy metabolism [25]. It was reported that myocardial ischemia and hypoxia lead to glucose metabolism disorders because glucose metabolism requires adequate oxygen [26], and 80% prevalence of coronary artery disease in patients with abnormal glucose metabolism in China [27]. The present results suggested an association of heart failure with qi deficiency with a glucose metabolism disorder. A low glucose metabolism level might serve as a biomarker of qi deficiency. Because of the ischemia and hypoxia of the body, energy metabolism decreased in cells, which resulted in the increase of citric acid in plasma. The cAMP level in plasma increased significantly which subjected to ischemia and hypoxia related to the mobilization of energy reserves under stress. Fatty acid oxidation became the major mode of energy supply when glucose metabolism disordered. This type of fat-related myocardial metabolism disorder not only increased ATP consumption and decreased ATP synthesis, but also increased myocardial oxygen consumption, which accelerates the process of cardiac dysfunction [28]. At day 45 after operation, the rats with qi deficiency were suffering from aggravated heart dysfunction, with significantly decreased citric acid, and the decrease of relevant metabolites reflected the metabolic failure of glucose, fat, and protein, and coincided with the reduced movement, aggregation and changes of respiratory rate in model rats. Moreover, the identical changes trend of Alanine and L-Valine on 4 and 45 day suggested that it is possible to excite the same syndrome at different time point.

During 7 ~ 28 days after the operation, the syndrome of blood stasis appeared in the qi deficiency rats with behavioral stress; colorimetric change of auricle, plantar and tongue; and increased prominence of peripheral circulatory disorders of blood stasis. The plasma metabonomics at 21 days after the operation showed a high level of citric acid, glucose, amino acids, and fatty acids when compare to the sham group, which indicated full mobilization of energy reserves. The increased catecholamine indicated the activation of sympathetic adrenergic system, which leading to the activation of protein lipase activity and increased endogenous lipolysis, so the high level of free fatty acids was detected [29]. The blood metabolic changes of glucopyranoside, dodecanoic acid, hexadecanoic acid and octadecanoic acid at 21 days showed the same trends with the metabolites of clinical patients with blood stasis syndrome in our previous study [9].

Interestingly, lipid metabolism disorder was detected at all of the three time points, even though the animals were not given a high-fat diet and did not receive other lipid interventions. At day 4 after the operation, the increases of dodecanoic acid, hexadecanoic acid and octadecanoic acid were detected. At day 21 after the operation, the changes of butanoic acid, propanonic acid, acetic acid and mononolinoleoylglycerol were observed. In particular, the decrease of mononolinoleoylglycerol might lead to the alterations of triglyceride level, cholesterol metabolism and lipid deposition, which match the necessary conditions for the development of coronary atherosclerosis. With the evolution of the syndrome, the status of the lipid metabolism disorder at the 45 day after the operation became relatively stable when compared to 21 day, indicating that continued abnormal lipid metabolism was associated with the evolution of ischemic syndromes.

Moreover, 14 metabolites were detected in all of the 3 time points, but without the same change trend, which might associated with the evolution of syndromes.

This study had several limitations. First, only one time point was analyzed in the same syndrome in this research. Second, because there were no published reports on the metabonomics of qi deficiency patients, the qi deficiency characters in this animal model need further verification.

## Conclusions

The Chinese medicine syndrome evolution was “qi deficiency-qi deficiency and blood stasis-qi deficiency” in an LAD ligation-induced model of HF in rats from 4 ~ 60 day after operation. The time course plasma metabonomics analysis provides evidence for the syndrome evolution. The QDBS and non-QDBS syndrome, as well as the qi deficiency and non-qi deficiency, could be well recognized through the plasma metabonomics analysis based on the spectral analysis data.

## Competing interests

The authors declare that there are no conflicts of interest regarding the publication of this article.

## Authors’ contributions

WW and LY developed the idea and designed the research. QQ wrote the manuscript, developed the search strategy, and ran the search with LCh, selected which studies to include and extracted the data from the studies with WY, interpreted the analysis and drafted the final review. XC obtained copies of the studies and revised the writing. LY carried out the analysis of PCA. All the authors read and approved the final manuscript.

## Pre-publication history

The pre-publication history for this paper can be accessed here:

http://www.biomedcentral.com/1472-6882/14/232/prepub

## Supplementary Material

Additional file 1: Table S1 **Syndrome diagnose indexes in MI rats.** To evaluate the rat HF model, ECG, physical signs, echocardiography, colorimetric analysis of images of the auricle, plantar and tongue, and blood studies were conducted. The results are shown in Additional file 1: Table S1. Click here for file
