# Supplementary material for: Plasma metabonomics study on Chinese medicine syndrome evolution of heart failure rats caused by LAD ligation
Source: BMC Complement Altern Med. 2014 Jul 9;14:232. doi: 10.1186/1472-6882-14-232 (PMC4227006; doi:10.1186/1472-6882-14-232)
Supplement: Additional file 1: Table S1 — Syndrome diagnose indexes in MI rats. To evaluate the rat HF model, ECG, physical signs, echocardiography, colorimetric analysis of images of the auricle, plantar and tongue, and blood studies were conducted. The results are shown in Additional file 1: Table S1. [file 1472-6882-14-232-S1.doc]

Table S1: Syndrome diagnose indexes in MI rats

|  | 4d | | 7d | | 14d | | 21d | | 28d | | 45d | | 60d | |
| --- | --- | --- | --- | --- | --- | --- | --- | --- | --- | --- | --- | --- | --- | --- |
|  | Model group | Sham group | Model group | Sham group | Model group | Sham group | Model group | Sham group | Model group | Sham group | Model group | Sham group | Model group | Sham group |
| Body weight | 247.40±24.36 | 239.17±18.40 | 254.27±12.17 | 245.75±13.25 | 268.11±8.87 | 264.85±18.54 | 282.60±14.13 | 282.83±21.49 | 294.61±18.81 | 295.87±17.89 | 332.29±60.29 | 344.48±39.72 | 363.36±90.16 | 383.53±53.35 |
| respiratory rate | 123.62±28.35* | 142.45±31.44 | 122.96±15.69* | 132.86±24.34 | 118.72±14.84* | 136.98±18.52 | 108.24±13.92* | 140.69±14.75 | 103.23±10.26* | 138.25±13.64 | 95.47±12.58** | 121.75±10.39 | 96.09±14.22** | 116.24±14.63 |
| LVEF | 0.34±0.09** | 0.93±0.02 | 0.57±0.06﹡ | 0.87±0.01 | 0.41±0.03** | 0.90±0.02 | 0.35±0.03** | 0.82±0.03 | 0.34±0.02** | 0.80±0.02 | 0.38±0.03** | 0.82±0..01 | 0.42±0.03** | 0.84±0.02 |
| LVFS | 0.14±0.04** | 0.60±0.06 | 0.24±0.03** | 0.50±0.01 | 0.17±0.0.1** | 0.56±0.02 | 0.14±0.01** | 0.46±-0.03 | 0.14±0.01** | 0.41±0.02 | 0.16±0.02** | 0.45±0.02 | 0.19±0.02** | 0.48±0.03 |
| PCV% | 42.73±1.78 | 41.09±2.41 | 42.22±1.84 | 41.43±2.59 | 42.53±2.72* | 39.99±2.24 | 45.38±1.96** | 41.5±2.12 | 53.31±3.78* | 48.28±2.79 | 48.84±2.50 | 47.36±2.41 | 47.56±1.82 | 46.39±2.29 |
| FIB% | 3.32±0.16 | 3.55±0.44 | 4.50±0.38 | 4.59±0.34 | 3.84±0.67 | 4.17±0.34 | 3.37±0.18 | 3.44±0.38 | 3.49±0.23 | 3.63±0.34 | 3.68±0.37 | 3.72±0.45 | 3.78±0.43 | 3.35±0.34 |
| PV mpa.s | 1.03±0.02 | 1.06±0.04 | 1.14±0.03 | 1.13±0.04 | 1.11±0.07 | 1.12±0.03 | 1.22±0.04 | 1.18±0.04 | 1.22±0.04 | 1.23±0.03 | 1.25±0.08 | 1.26±0.07 | 1.25±0.06 | 1.24±0.09 |
| PA% | 64.65±16.41 | 62.08±12.89 | 49.35±6.74** | 37.83±9.94 | 58.19±7.57* | 48.0±10.44 | 43.28±3.94 | 48.66±5.43 | 55.16±8.78 | 48.57±6.39 | 44.77±7.41 | 48.49±8.08 | 47.90±5.66 | 47.42±7.46 |
| AV 10S-1 | 6.82±0.76 | 6.48±0.86 | 6.48±0.38 | 5.95±0.83 | 6.40±0.81* | 5.55±0.66 | 6.61±0.57** | 5.42±0.48 | 8.13±1.34* | 6.90±0.67 | 7.64±0.73 | 7.11±0.67 | 7.84±0.99 | 7.53±0.58 |
| AV 50S-1 | 4.29±0.49 | 4.11±0.38 | 4.38±0.13* | 4.06±0.41 | 4.08±0.35* | 3.74±0.38 | 4.53±0.22** | 3.96±0.26 | 4.79±0.52 | 4.35±0.27 | 4.74±0.41 | 4.49±0.33 | 4.96±0.54 | 4.85±0.30 |
| AV 200S-1 | 3.24±0.26 | 3.19±0.17 | 3.29±0.07** | 3.07±0.21 | 3.18±0.19 | 3.04±0.21 | 3.70±0.13** | 3.36±0.20 | 3.63±0.38 | 3.31±0.13 | 3.48±0.21 | 3.33±0.18 | 3.62±0.28 | 3.66±0.41 |
| RV 10S-1 | 14.32±0.63 | 13.84±0.86 | 14.46±0.59* | 13.22±1.69 | 12.65±1.28* | 11.36±1.29 | 12.33±0.86** | 10.64±0.77 | 13.32±1.65 | 12.2±0.94 | 13.63±1.64 | 12.88±0.96 | 14.39±2.10 | 14.07±1.01 |
| RV 50S-1 | 7.94±0.23 | 7.58±0.82 | 8.02±0.30* | 7.39±0.79 | 7.23±0.67 | 6.85±0.82 | 7.79±0.37* | 7.11±0.42 | 7.08±0.56 | 6.94±0.43 | 7.69±1.00 | 7.39±0.59 | 8.35±1.21 | 8.31±0.65 |
| RV 200S-1 | 6.02±0.31 | 5.83±0.42 | 6.04±0.27 | 5.56±0.50 | 5.14±0.46 | 5.13±0.46 | 5.96±0.30 | 5.68±0.26 | 4.91±0.38 | 4.77±0.20 | 5.11±0.49 | 4.92±0.39 | 5.52±0.68 | 5.73±0.78 |
| auricle R | 198.27±8.32 | 196.33±6.73 | 197.03±7.91 | 197.229±4.29 | 193.38±8.36 | 194.82±5.88 | 193.15±6.02 | 197.51±7.05 | 192.64±11.64 | 186.81±5.28 | 195.85±6.31 | 198.87±3.75 | 204.20±30.12 | 193.627±8.17 |
| auricle G | 139.42±13.21 | 136.94±8.64 | 138.11±12.43 | 137.59±9.00 | 141.05±10.11 | 140.14±8.72 | 130.38±7.52* | 138.02±9.32 | 132.81±14.62 | 125.59±11.89 | 137.32±8.48 | 141.66±3.38 | 139.43±5.58 | 142.50±9.92 |
| auricle B | 118.49±10.33 | 118.06±7.94 | 112.45±12.07 | 116.08±9.51 | 122.49±11.87 | 122.48±7.98 | 110.33±9.60 | 117.29±12.23 | 119.39±14.68 | 110.92±12.01 | 120.82±8.38 | 124.49±2.83 | 121.68±4.79 | 126.08±10.20 |
| plantar R | 169.84±3.82 | 172.44±2.08 | 176.29±4.96* | 182.04±6.35 | 183.74±4.13** | 192.63±7.82 | 177.89±6.01* | 184.44±4.71 | 169.19±10.29** | 185.99±7.61 | 192.71±6.11 | 193.61±7.47 | 193.71±3.99 | 194.51±3.75 |
| plantar G | 142.92±5.30 | 145.37±4.28 | 140.40±4.20 | 145.35±7.06 | 146.39±5.76** | 154.37±8.81 | 142.48±5.61* | 148.41±5.16 | 136.63±9.35* | 147.65±8.04 | 157.64±6.50 | 160.64±8.73 | 157.88±7.87 | 159.80±5.66 |
| plantar B | 131.26±4.20 | 134.35±9.42 | 128.36±3.86 | 133.20±8.98 | 133.84±7.03** | 143.90±9.30 | 131.95±5.38* | 137.19±6.43 | 126.02±9.31* | 136.55±9.30 | 145.58±7.62 | 148.83±9.59 | 146.76±8.29 | 147.82±5.16 |
| tongue R | 157.62±5.73 | 158.01±7.20 | 165.65±3.35 | 167.05±5.06 | 154.73±6.03** | 162.08±6.05 | 155.95±7.94 | 161.49±5.86 | 149.55±9.90 | 150.78±7.33 | 160.67±7.01 | 164.47±4.73 | 162.22±5.29 | 163.52±5.48 |
| tongue G | 103.26±5.33 | 104.39±7.43 | 114.07±4.66 | 115.45±5.14 | 105.28±6.62 | 108.39±7.5 | 100.78±7.90 | 106.63±7.46 | 101.95±4.03 | 96.08±8.00 | 109.07±14.47 | 109.98±2.52 | 105.48±6.55 | 104.69±5.23 |
| tongue B | 100.84±5.12 | 97.41±4.56 | 103.22±4.14 | 102.48±5.98 | 92.80±6.61 | 95.38±8.57 | 91.59±7.03 | 96.34±6.85 | 93.70±4.79** | 85.8±6.60 | 99.48±13.28 | 100.96±4.21 | 95.97±6.50 | 94.88±5.40 |

Note：* P<0.05 compare with the Sham Group, ** P<0.01 compare with the Sham Group
